# Impact of respiration on stroke volumes in paediatric controls and Fontan patients assessed by MR real-time phase velocity mapping

**DOI:** 10.1186/1532-429X-16-S1-P107

**Published:** 2014-01-16

**Authors:** Kai T Laser, Peter Barth, Andrea Kelter-Kloepping, Juergen Gieseke, Wolfgang Burchert, Hermann P Esdorn, Hermann Koerperich

**Affiliations:** 1 HDZ-NRW, Center for Congenital Heart Defects, Bad Oeynhausen, Germany; 2 HDZ-NRW, Radiology, Nuclear Medicine and Molecular Imaging, Bad Oeynhausen, Germany; 3 Philips Healthcare, Hamburg, Germany; 4 Klinik Bad Oexen, Bad Oeynhausen, Germany

## Background

Routine blood volume quantification is generally performed by using two-dimensional velocity-encoded phase-contrast magnetic resonance imaging (PC-MRI). Due to their long acquisition times averaging of flow information occur leading to underestimation of peak velocities and preventing the examination of respiration-related stroke volume (SV) variations.

## Methods

Non-electrocardiographic triggered real-time PC-MRI using EPI combined with half-Fourier technique (TR/TEeff/excitation angle = 12-14 ms/3.3 ms/40°, SENSE-factor = 4, temporal resolution = 24-28 ms) was applied to study respiration-driven SV fluctuations in the ascending aorta (AAo) and both caval veins (SVC, IVC) under natural and forced breathing in 34 healthy children and 10 pediatric Fontan patients. Data were collected during a 12-14 s time interval. The subject's physiological data (ECG & respiration) were recorded simultaneously for matching with flow data. Virtual stroke volumes were generated dividing the respiration curve in four segments: expiration, breathing in, inspiration and breathing out. Spirometric measurements were performed to validate respiratory volumes. Statistical differences were analyzed by using ANOVA, paired Student t-test and Bland-Altman statistics.

## Results

Stroke volume variability in healthy subjects was around 6% for AAo and >15% for SVC and IVC while breathing naturally. Under forced breathing variability increased to 9% and >34%, respectively. In Fontan patients a two to three-fold augmentation was observed in both respiratory maneuvers. Whereas in volunteers aortic SV was elevated during expiration (6%) and decreased during inspiration (-6%) in relation to mean respiratory SV, highest blood flow was detected during breathing in SVC (10%) and IVC (20%) and lowest blood flow during breathing out (-12%, -15%). Differences were increased under forced breathing. All differences were statistically significant. Regarding patients SV variability was drastically increased and had to be related to the patient's individual quality of Fontan circulation. Intraobserver and interobserver varability of the four separated respiration-dependent SV was below 5%.

## Conclusions

Due to its non-averaging character real-time PC-MRI allows a physiological assessment of respiratory-related SV fluctuations in healthy subjects as well as in Fontan patients and demonstrates its capability for detection of short-term effects in clinical routine work. It can be expected that real-time PC-MRI has the potential to classify the quality of Fontan hemodynamics.

## Funding

None.

